# The Disputes between the Editors of the Dental Journals

**Published:** 1889-12

**Authors:** J. A. Robinson

**Affiliations:** Jackson


### The Disputes Between the Editors of the Dental Journals.

There were three boys of us in a small New England home whose names were Frank, Jerie and Bill; sometimes in doing the chores of the family, we had little bickerings and disputes about the work as to which should bring in the wood, or draw the water, or run to the store on some little errand, or who had done the most work. At such times mother, who was a very practical woman, would make us all sit down on little crickets (we had no little chairs) and repeat aloud and in unison after her, from Dr. Watts' Hymns for Infant Minds, this verse:

"The wise will let his anger cool, at least before 'tis night,
But in the bosom of a fool, it burns 'till morning light."

and then say "Now, boys, go to your work and let me hear no more noise."

The three dental journals, the *Cosmos*, the oldest; *The Items of Interest*, the next oldest, and the *International*, which is the youngest, reminds us of that family of boys in the disputes it has in its work in the dental profession.

The three journals are doing good work, and each one is trying to realize its ideal; they all give us facts of real value because they express great and fruitful truths.

Suppose *The Items of Interest* does copy articles from other journals without giving credit for them, as is charged; he only sows the ideas broadcast before the brothers, and gives them away for one dollar or gives them free; and they enlighten some poor fellow that the other journals do not reach, for the reason that many of the other journals publish so many heavy articles that are above the comprehension of the average dentist of to-day; articles that the average dentist is not prepared to read.

Many a practical operator who understands the therapeutics of dentistry, and is well up in mechanics, in practical, prosthetic and operative dentistry, does not care for histology and would not read it if placed before him all his days, as he is within limitations that he can never surmount. He is limited by birth and education and endowments; he is like the string of the harp that is tied at both ends, and the distance and tension will not allow of a higher or lower sound, and yet he is a useful and faithful brother and good citizen, and the sound he makes is necessary to the full and perfect harmony of the music, and for such a person to attempt anything more, he would accomplish less. There is always a certain class of would-be scholars who do nothing but criticize every scheme that looks to the general elevation of the masses, and who, with all their learning, do less for the cause of humanity than those with less learning and more wisdom. There are only a few persons in the professions or the sciences who do advance work, and those persons are generally attached to colleges or have fixed incomes. There has never been but one Darwin or Tyndale, never but one Newton or Huxley. In this hurrying world of living getting, it is impossible; besides if a person is in advance of the crowd, he is an enthusiast and is looked upon as a crank or a fool. We do not wish to be understood as disparaging education. The educated class are the generals of the army, but it takes the under officers and common soldiers to fight the battles and win the victories.

The last verse of Dr. Watts' hymn our mother made us repeat runs like this:

> "Pardon, O Lord, our childish rage,
> Our wicked thoughts remove;
> So as we grow to riper age
> Our hearts may grow in love."

Perhaps our brother editors had better learn and repeat Dr. Watts' hymn. J. A. Robinson.

Jackson, November 15, 1889.

